# Effects of iTBS-rTMS on the Behavioral Phenotype of a Rat Model of Maternal Immune Activation

**DOI:** 10.3389/fnbeh.2021.670699

**Published:** 2021-04-21

**Authors:** Nadine Rittweger, Tanja Ishorst, Gleb Barmashenko, Verena Aliane, Christine Winter, Klaus Funke

**Affiliations:** ^1^Department of Neurophysiology, Medical Faculty, Ruhr-University, Bochum, Germany; ^2^AIO-Studien-gGmbH, Berlin, Germany; ^3^Department of Psychiatry and Psychotherapy, Charité University Medicine Berlin, Berlin, Germany; ^4^Department of Psychiatry and Psychotherapy, Medical Faculty Carl Gustav Carus, Technische Universität Dresden, Dresden, Germany

**Keywords:** maternal immune stimulation, schizophrenia, animal model, behavioral phenotypes, rTMS, iTBS, sham stimulation, history of experience

## Abstract

Repetitive transcranial magnetic stimulation (rTMS) is considered a promising therapeutic tool for treating neuropsychiatric diseases. Previously, we found intermittent theta-burst stimulation (iTBS) rTMS to be most effective in modulating cortical excitation-inhibition balance in rats, accompanied by improved cortical sensory processing and sensory learning performance. Using an animal schizophrenia model based on maternal immune activation (MIA) we tested if iTBS applied to either adult or juvenile rats can affect the behavioral phenotype in a therapeutic or preventive manner, respectively. In a sham-controlled fashion, iTBS effects in MIA rats were compared with rats receiving vehicle NaCl injection instead of the synthetic viral strand. Prior to iTBS, adult MIA rats showed deficits in sensory gating, as tested with prepulse inhibition (PPI) of the acoustic startle reflex, and deficits in novel object recognition (NOR). No differences between MIA and control rats were evident with regard to signs of anxiety, anhedonia and depression but MIA rats were somewhat superior to controls during the training phase of Morris Water Maze (MWM) test. MIA but not control rats significantly improved in PPI following iTBS at adulthood but without significant differences between verum and sham application. If applied during adolescence, verum but not sham-iTBS improved NOR at adulthood but no difference in PPI was evident in rats treated either with sham or verum-iTBS. MIA and control rat responses to sham-iTBS applied at adulthood differed remarkably, indicating a different physiological reaction to the experimental experiences. Although verum-iTBS was not superior to sham-iTBS, MIA rats seemed to benefit from the treatment procedure in general, since differences—in relation to control rats declined or disappeared. Even if classical placebo effects can be excluded, motor or cognitive challenges or the entire handling procedure during the experiments appear to alleviate the behavioral impairments of MIA rats.

## Introduction

Schizophrenia is considered a neurodevelopmental disorder originating from disturbed neuronal maturation at prenatal stage and/or during adolescence (Insel, 2010; Selemon and Zecevic, 2015). Epidemiological studies demonstrate a relationship between infections during pregnancy and increased risk of the offspring to develop a schizophrenic phenotype during early adulthood (Mednick et al., 1988; Brown et al., 2004; Brown, 2012; Estes and McAllister, 2016). Based on these findings rodent maternal immune activation (MIA) models have been launched which use injections of either viral or bacterial pathogens to pregnant dams at a particular state of gestation (Zuckerman et al., 2003; Zuckerman and Weiner, 2005; Meyer, 2014; for review see Bergdolt and Dunaevsky, 2019).

Converging evidence obtained from patient studies and MIA models suggests aberrant synchrony of long-range neuronal network oscillations as a major cause of psychotic states and cognitive deficits as observed in schizophrenia and other psychiatric disorders (for reviews see Sukhodolsky et al., 2007; Lisman and Buzsáki, 2008; Uhlhaas and Singer, 2010; Başar, 2013). Disturbed local and long-range synchronization of neuronal activity is likely a result of maldevelopment of neurons and/or their connections (Insel, 2010; Selemon and Zecevic, 2015) leading to a misbalance of excitatory and inhibitory processes, most strikingly evidenced by a reduced function of the interneurons expressing the calcium-binding protein parvalbumin (PV; for review of knowledge obtained from clinical studies and animal models see Lewis et al., 2012; Ferguson and Gao, 2018).

Pharmacological treatment of schizophrenia in the adult is still unsatisfying since alleviation is mostly restricted to the positive symptoms of the disease but less improves the cognitive deficits. And, often a pharmacoresistance develops due to a plastic response of the neuronal system, e.g., changes in the number of receptors targeted by the drug, further limiting the efficiency of drug treatment. Furthermore, neuronal malfunctions resulting from a disturbed development are even more difficult to treat at adult state. Currently, preventive interventions in the pharmacological, social and cognitive-behavioral regime are discussed (Reisinger et al., 2015; Millan et al., 2016). As non-invasive brain stimulation (NIBS) techniques, like transcranial direct current stimulation (tDCS) and repetitive transcranial magnetic stimulation (rTMS), have been shown to modulate cortical excitability and plasticity (Huang et al., 2005; Ziemann and Siebner, 2008; Ridding and Ziemann, 2010; Dayan et al., 2013), they may also be considered as alternative therapeutic or possible preventive tools (Post and Keck, 2001; Padberg and George, 2009; Rajji et al., 2013; Kuo et al., 2017; Iimori et al., 2019; Hadar et al., 2020) in the context of schizophrenia (Hadar et al., 2018, 2020).

We previously demonstrated that rTMS, and particularly the intermittent theta-burst stimulation (iTBS; Huang et al., 2005) protocol, is effective in modulating cortical excitability in rats. In this line, iTBS reduced expression of inhibitory activity markers like GAD67, PV and calbindin (CB; Trippe et al., 2009; Benali et al., 2011), increased evoked sensory responses (Thimm and Funke, 2015), and improved tactile associative learning (Mix et al., 2010). In a recent study we could further demonstrate that iTBS is able to alleviate aberrant synchrony of oscillatory brain activity within the limbic system of MIA offspring (Lippmann et al., 2021).

To evaluate its therapeutic and preventive potential, we applied iTBS either to adult or juvenile MIA rats, respectively, and tested the rats with regard to changes in behavioral phenotypes possibly induced by MIA, like deficits in attentional directing or spatial orientation/learning, or depression- and anxiety-like behaviors. With regard to the findings of previous experiments showing iTBS to modulate molecular and electrical neuronal activity markers as well as behavior, as mentioned above, we expected iTBS to have either a beneficial or detrimental effect on behavioral performance, depending on how it modulates inhibitory cortical activity and that application during adolescence has more profound effects. According to the effects of MIA, we expected different iTBS effects compared to controls. Given the many reports on sham-stimulation effects in humans we further expected to find additional effects related to testing and treatment, in particular in the groups of adult rats being re-tested, although pure placebo effects can be excluded. We found iTBS to reduce some of the behavioral deficits of MIA rats, like with sensory gating and novel object recognition (NOR), however, the effects of verum stimulation were largely not superior to sham stimulation, indicating a general beneficial effect of the cognitive and motor activity related to the testing and handling procedure. Interestingly, sham effects were different in MIA and control rats indicating differences in the processing of experiences related to the experimental conditions.

## Materials and Methods

### Animals

Pregnant Wistar rats were delivered by Charles-River (Sulzfeld, Germany) on gestational day (GD) 13 for the purpose of MIA at GD15. Prior to iTBS and behavioral testing the dams and their offspring were housed within the central experimental animal facility of the medical faculty with free access to food pellets (V1534-000, Ssniff Spezialdiäten GmbH, Soest, Germany) and tap water and with a light-dark cycle of 12/12 h (light on at 6 am). On postnatal day 21, offspring were separated from their mothers with females and males housed separately in groups of four per cage (Macrolon type IV). To avoid additional variability due to varying hormone status of the females and for better comparability to other NIBS studies on male MIA offspring, we included only males in this study. One week before starting the experimental procedures the rats were moved to ventilated cabinets within the department and randomly allocated to the different experimental groups (see below) while still housed in the same groups of 4 animals per cage (Macrolon type IV). All experiments were performed in compliance with German laws and the directive of the European Community (2010/63/EU, Sept. 22th, 2010) for the use of animals in research and were approved by the local ethics committee (State Office for Nature, Environment and Consumer Protection, LANUV, Section 81-Animal Welfare, Az. 84-02.04.2014.A294).

### Experimental Groups

We conducted two experimental series with sham or verum-iTBS applied to MIA offspring (termed MIA rats in the following) and age-matched controls either at adulthood (3–4 months old, *Exp. I*), or during adolescence (6 weeks old, *Exp. II*). To evaluate a possible therapeutic effect of iTBS (*Exp. I*) the behavioral phenotype of the adult rats was determined before and after stimulation. To test a possible preventive action, stimulation was applied during adolescence and behavioral testing followed when rats were adult. The offspring of each litter (MIA or controls) were randomly attributed to these experimental series and subgroups (sham or verum-iTBS) by a technician to achieve blinding, with each group composed of offspring originating from five different MIA and five different control litters.

### Induction of MIA

To induce MIA, polyinosinic:polycytidylic acid (PolyI:C, 4 mg/kg, Sigma–Aldrich P1530, Steinheim, Germany dissolved in 1 ml 0.9% sterile NaCl) was injected in the tail vein at GD 15 as has been done in previous studies (see Hadar et al., 2020). To avoid stress and to enable a safe and precise injection, rats were transiently sedated by placing them within a desiccator equipped with an isoflurane (Forene^®^ Abbvie GmbH, Ludwigshafen, Germany) soaked sponge. Control animals received 1 ml/kg NaCl vehicle in the same way.

### rTMS (iTBS)

iTBS was applied to conscious rats as previously described in more detail (e.g., Mix et al., 2014; Kloosterboer and Funke, 2019): after the animals had been adapted to the handling (manual restrain) and the noise and skin sensations of iTBS over a period of about a week, daily iTBS (Monday to Friday) was applied using a Magstim rapid^2^ and a 2 × 70 mm figure-of-eight coil (Magstim Limited, Whitland, Dyfed, UK). In a manner of accelerated rTMS (for review see Sonmez et al., 2019), each rat received three iTBS blocks of 600 pulses per day at 15–20 min intervals (1,800 pulses/day). This inter-block interval was chosen because it had been shown to amplify molecular iTBS effects in rat models (Volz et al., 2013) and effects on motor cortex in humans (Nettekoven et al., 2014). One iTBS block consisted of 20 trains, with each train consisting of 10 bursts (three pulses @ 50 Hz) repeated at 5 Hz. The trains were applied at intervals of 10 s (2 s ON/8 s OFF). Thus, one block lasted 192 s in total and was well tolerated by the animals without obvious discomfort or extensive movements. With regard to the fast maturation of rats and because of the higher succeptability of the juvenile brain to plasticity-inducing stimulation procedures we applied iTBS sessions only at 10 days (2 weeks) to the juvenile rats but stimulated the adult rats at 20 days (4 weeks).

Stimulus intensity was set to 21–23% of maximal machine output for the adult rats as done in the previous studies. In case of the juvenile rats, stimulus intensity had to be increased to 30% due to the smaller brain size (Weissman et al., 1992) to achieve comparable stimulation efficiency as estimated by induced muscle twitches. Finally, for each individual animal the distance between coil and head of the animals varied between 5 and 10 mm to determine the optimal position to just prevent activation of body and limbs muscle, thus stimulation strength was below motor threshold. Stimulation intensity was re-adjusted if muscle twitches occurred at a coil-to-brain distance closer as 10 mm. The coil was placed in a way to induce a mediolateral oriented electric field suitable to activate cortical areas *via* the callosal axons while preventing stimulation of deeper structures (see Kloosterboer and Funke, 2019; Murphy et al., 2016, for more details). In case of sham stimulation the coil was lifted by 10 cm to prevent magnetic stimulation but exposing the animal to the sound of the TMS coil while manually restraining it in the same way.

### Behavioral Testing Procedures

#### Prepulse Inhibition (PPI)

Prepulse Inhibition (PPI) test is based on the acoustic startle response (ASR) and estimates sensory gating by applying a prepulse of lower intensity (69, 73 and 83 dB) 100 ms prior to the startle stimulus (100 dB) (Swerdlow et al., 2008). PPI of the ASR was measured in a sound-shielded chamber equipped with a small mesh-wire cage (220 × 90 × 90 mm) mounted on a motion-sensitive transducer platform (TSE, Bad Homburg, Germany) to register the strength of the rat’s flinch response. All sounds had durations of 20 ms and were applied *via* two loudspeakers as broad-band (white) noise signals on a continuous noise level of 60 dB SPL. Following acclimatization for 5 min, first a sequence of 10 startle stimuli (100 dB) was applied in isolation. Then, during the testing phase either the startle stimulus alone, one of the three pre-pulses alone, or a combination of one of the three pre-pulses with the startle pulse were quasi-randomly applied 10 times *via* custom software control. Stimuli were applied at intervals of 25 s with a jitter of ±5 s. PPI was calculated as 100 − mean prepulse-startle response/mean startle response separately, for each prepulse intensity.

### Sucrose Consumption Test (SCT)

The Sucrose Consumption Test (SCT) is used as a measure of anhedonia if rats show a decreased preference of sweet solution over tap water (decreased ability to experience pleasure and reward; Papp et al., 1991). Rats were first habituated to the sweet test solution (Nestlé, Milchmädchen gezuckerte Kondensmilch, 1:3 diluted with tap water) and adapted to the test cage and the bottle two days prior to testing. One day before testing, rats were restricted to 15 g food pellets per animal but with water *ad libitum*. On the test day itself rats had free access to the test solution for 15 min. The amount of consumed solution was determined by weighting the bottles before and after testing and by normalization to the individual body weight prior to testing.

#### Elevated Plus Maze (EPM)

Elevated plus maze (EPM) is a test for anxiety and determines how long rats stay on an open arm of a cross-shaped maze compared to arms enclosed by walls (Pellow et al., 1985). Each arm had a length of 90 cm and a width of 20 cm for the open arms and 8 cm for the closed. Two opposing arms were equipped with walls of 19 cm height starting at 10 cm from the center of the cross. The maze was placed in a brightly illuminated room at a height of 62 cm from bottom. The 5 min procedure which was video-recorded from top was started by placing the rat at the center of the maze with the head facing one of the open arms in a random order. Percent of time the rat spent in one of the open arms (full body out of the walls) was determined off-line by video analysis (Pinnacle Studio 10.6). Rats staying all the time at the central platform without moving to the open or closed arms were excluded from analysis.

#### Novel Object Recognition (NOR)

This procedure tests if rats are aware of either novel objects or changed places of objects (Ennaceur and Delacour, 1988). We tested for the recognition of novel objects 1 and 24 h after a previous configuration. Prior to the testing, rats were familiarized with the testing box (85 × 85 cm, 45 cm height) for 45 min. Three of four different objects were used in a random order. The acrylic objects were 20 cm in height and about 10 cm in diameter and had a different shapes (cylindrical, quadratic, triangular and hexagonal) and colors (red, yellow, blue, green inlays). The position of the objects to be replaced by a new object was changed from rat to rat to exclude place preferences according to room landmarks. After the two objects were placed equidistantly from the walls within the test arena, a rat was placed between the two objects facing one wall. The rat was allowed to explore the objects for 5 min while the process was video-recorded. The time the rat inspected each object with the criterion that at least one whisker or another part of the body was in close contact with the object was determined by off-line video analysis. A preference index (PI) was calculated as the ratio of the difference between time spent for new and old object to the sum of both [(T_new_ − T_old_)/(T_new +_ T_old_)] yielding a range of −1 (only old object) to +1 (only new object).

#### Morris Water Maze (MWM)

The test was conducted according to Terry et al. (2011). A pool of 180 cm in diameter and 60 cm height was filled to a height of 30 cm with water at 22°C. For the purpose of video tracking/analysis, we have chosen a black pool to achieve a better contrast to the white body of the rats. The platform (12 cm in diameter) was made of transparent acryl to obtain invisibility if hidden by the water. When raised above water level during first trial, the platform was equipped by a bright signal red circumference to enhance visibility against transparent water by the rat. The pool was virtually divided into four cardinal sectors and the platform area. Automatic tracing of the rat’s position was achieved by custom software video analysis based on the open source routines provided by Aguiar et al. (2007). The position of the white body of the rat against the black background can be reliably traced if preventing light reflections on the water surface, e.g., by using indirect illumination of the room. In addition to landmarks of the room, the walls of the pool were equipped with four different high contrast patterns at each cardinal direction.

MWM training sessions of four trials each were performed on four subsequent days while final place memory test happened on the 5th day with the platform removed. The platform was always located in the middle of the north quadrant, equidistantly from the wall and the center of the basin (45 cm). Only in case the MWM was performed a second time (rats having received iTBS in adult state) the platform location was switched to the south quadrant. The platform was above water level and thus visible for the rats for the four trials on day 1, but hidden below water surface on days 2–4. The platform was removed for the final memory test on day 5. For each of the four trials of one session the rat was released at a different quadrant facing the wall of the basin. The order of quadrants was randomized for each session. Rats were given a relaxing phase between trials (30–60 min) by testing the rats in an interleaved fashion. After each trial, rats were dried by a towel and placed in a cage under a red heat lamp. Each trial lasted for 90 s and the rat was guided by hand to the platform if not hitting the platform within this time. After the rat climbed the platform it was allowed to visually explore and memories the environment before being put back in the cage. The path of the rat was continuously tracked by the software and used to calculate the following parameters: total time to reach the platform, time spent in each quadrant (incl. platform area) and the number of entries to each quadrant online. Offline analysis further included total path length, mean swimming speed and mean distance from platform and pool center to further calculate five behavioral types (1 = thigmotaxis, 2 = cycling, 3 = random, 4 = corrected and 5 = direct, see Illouz et al., 2016) with the value indicating worst (1) to best (5) grade. In case of the final memory trial (with the platform removed) percent of time the rat spent within each quadrant for the first 30 s was calculated and time spent in the target quadrant was set in relation to the non-target quadrants.

#### Porsolt Forced Swim Test (PFST)

The Porsolt Forced Swim Test (PFST) had been developed as a paradigm to test the efficiency of antidepressive substances in rodents. It measures the time the animals spends in actively trying to escape from the aversive situation (swimming, struggling) vs. passive behavior, with the latter interpreted as a sign of depression-like behavior (Porsolt et al., 1977). In the test rats were placed for 5 min in transparent acrylic cylinders of 52 cm height and 19 cm in diameter, filled with water at 22°C up to a level of 35 cm. The procedure was video-recorded from aside to determine off-line the time spent with struggling, swimming, diving and floating. Floating was classified as being “immobile” while the other behaviors were classified as “active.” Finally, the ratio of immobile to active behavior was calculated. The rats were immediately removed from the water if they showed signs of respiratory distress and near-drowning. Afterward, rats were dried with a towel and placed in a cage with red light warming.

### Statistical Analysis

All data sets were first tested for normal distribution using Shapiro–Wilk test. Two rats, one of the sham-iTBS control group and one of the MIA sham-iTBS group, did not move at all during EPM test. To avoid falsification of group means these data were excluded but imputated by group means. It turned out that all data sets appeared to be normally distributed after correction of these two outliers and were subjected to parametric tests. Two-factorial analysis of variance (ANOVA) using factors GROUP (MIA vs. Controls) and iTBS with either pre vs. post data for sham- and verum-iTBS in case of the adult rats, or sham vs. verum in case of the juvenile rats not tested before iTBS. Pairwise comparison of MIA vs. control groups and sham vs. verum groups was done using *t*-test for independent samples, while paired *t-test* was applied to compare pre- vs. post-iTBS data within a group. A difference was considered being statistically significant with *p* < 0.05. Partial eta^2^ (*η*^2^) was calculated as effect size in case of ANOVA while Cohen’s-d (*d*) was calculated for *t-test* results on the basis of common SD.

## Results

### Exp. Series I: iTBS Applied to Adult Rats (Therapeutic Approach)

Application of iTBS to adult MIA rats aimed at testing a possible therapeutic effect of this method. Therefore, the behavioral testing battery was conducted once before (data set pre-iTBS) and once after 4 weeks of daily iTBS (Monday–Friday, 3 blocks/day, data sets sham and verum-iTBS). This series was conducted in six blocks with each block consisting of two MIA offspring, with one receiving verum-iTBS, the other receiving sham-iTBS, and two corresponding controls (all male and from one litter).

#### PPI

**Figure 1:**
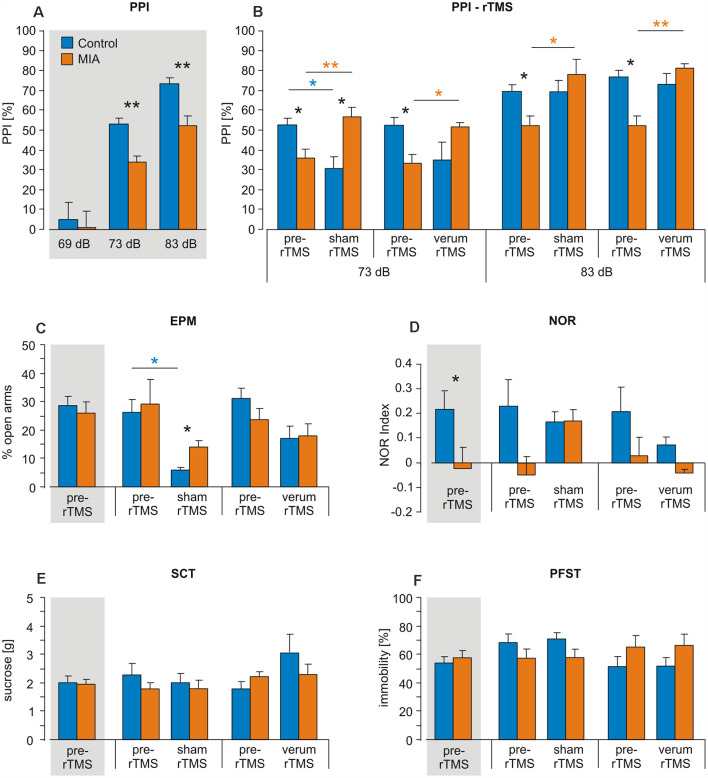
Intermittent theta-burst stimulation (iTBS) applied to adult rats: effects on prepulse inhibition (PPI), elevated plus maze (EPM), novel object recognition (NOR), sucrose consumption test (SCT) and porsolt forced swim test (PFST). Maternal immune activation (MIA) and control rats (*n* = 12 + 12) were tested for **(A,B)** PPI, **(C)** EPM, **(D)** NOR, **(E)** SCT and **(F)** PFST once before iTBS (pre-iTBS, gray shading: all pre-iTBS measures pooled) and a second time after either sham-iTBS or verum-iTBS (*n* = 6 per group). Colored asterisks above lines indicate statistically significant differences between pre- and post-iTBS (sham or verum) data determined by paired *t*-test (controls: blue, MIA: orange, here pre-iTBS data shown separately for sham and verum groups) while black asterisks indicate significant differences between MIA and control rats as revealed by *t*-test for independent samples (two-sided). *p < 0.05, **p < 0.01. Data are shown as group means ± SEM.

ANOVA conducted with factors GROUP (Controls, MIA) and PREPULSE (69, 73, 83 dB) for PPI measurements prior to iTBS revealed a significant effect of both factors (GROUP: *F*_(1, 71)_ = 10.364, *p* = 0.002, *η*^2^ = 0.136; PREPULSE: *F*_(2, 71)_ = 71.568, *p* < 0.001, *η*^2^ = 0.684) indicating not only significant differences in PPI as induced by different prepulse strength but also a difference in PPI between MIA rats and controls. *Post hoc* *t*-test (for independent samples) revealed significantly less PPI in MIA rats compared to controls in case of 73 and 83 dB prepulse (73 dB: *T*_(22)_ = 3.565, *p* = 0.0017; *d* = 1.46; 83 dB: *T*_(22)_ = 3.915, *p* < 0.001; *d* = 1.60; Figure 1A).

MIA and control rats were then split into two groups, with one receiving sham-iTBS and the other verum-iTBS. ANOVA conducted with factors GROUP (Controls, MIA) and iTBS (pre, post) revealed no significant effects of both factors when analyzing sham and verum-iTBS groups tested with 73 and 83 dB prepulse but a highly significant interaction of both factors in case of sham- and verum-iTBS when tested with 73 dB prepulse and with verum-iTBS when tested with 83 dB prepulse (for further details see Table 1). A comparison of post-iTBS to pre-iTBS PPI values using paired *t*-test revealed a significant increase in PPI for MIA rats after sham and verum-iTBS both, in case of 73 dB and 83 dB prepulse (see Table 1 and Figure 1B, orange asterisks). Differently, the controls showed a significant decrease in PPI after sham-iTBS when tested with 73 dB prepulse (Table 1, Figure 1B, blue asterisks). Post-iTBS data further showed a significant difference of PPI values between the sham-iTBS treated MIA and control rats (*T*_(10)_ = −2,710, *p* = 0.026, *d* = 1.34).

**Table 1:** Analysis of variance (ANOVA and *post hoc* *t*-test results of the analysis of prepulse inhibition (PPI) data in case of ntermittent theta-burst stimulation (iTBS) applied to adult rats, using factors Group (maternal immune activation, MIA, controls) and iTBS (pre, post).

	ANOVA					*t*-test pre vs. post iTBS (sign. cases)				
Parameter	Factor	DF	*F*-value	*p*-value	*η*^2^	Case	DF	*T*-value	*p*-value	*d*
Verum-iTBS
73 dB prepulse	Group	1		n.s.		MIA	5	−3.014	**0.03**	1.62
	Itbs	1		n.s.						
	Group × iTBS	1	6.522	**0.019**	0.246					
Sham-iTBS										
73 dB prepulse	Group	1		n.s.		MIA	5	−4.887	**0.005**	1.10
	iTBS	1		n.s.		controls	5	3.070	**0.028**	1.56
	Group × iTBS	1	9.812	**0.005**	0.329
Verum-iTBS
83 dB prepulse	Group	1		n.s.		MIA	5	−3.651	**0.015**	2.18
	iTBS	1		n.s.
	Group × iTBS	1	10.385	**0.004**	0.342
Sham-iTBS
83 dB prepulse	Group	1		n.s.		MIA	5	−3.913	**0.011**	1.26
	iTBS	1		n.s.
	Group × iTBS	1		n.s.

*DF, degrees of freedom; *η*^2^, partial ETA; d, Cohen’s d*; n.s. -, not significant; *p*-values of significant cases (<0.05) are shown in bold font.

#### EPM

MIA rats did not differ from Control rats with regard to percent time spent in the open arms during EPM test when tested prior to iTBS (*p* = 0.911; Figure 1C). ANOVA conducted with the factors GROUP (Controls, MIA) and iTBS (pre, post) after sham- or verum-iTBS had been applied revealed no significant effect of factor GROUP but a significant effect of factor iTBS in case of sham-iTBS (*F*_(1,25)_ = 5.824, *p* = 0.025, *η*^2^ = 0.209) and a strong trend in case of verum-iTBS (*F*_(1,25)_ = 3.569, *p* = 0.073, *η*^2^ = 0.151). Paired *t*-test yielded a significant decrease in open arm stays for the control rats after sham-iTBS (*T*_(6)_ = 2.495, *p* = 0.047, *d* = 1.17) but no significant difference after verum-iTBS. In case of MIA rats, neither sham- nor verum-iTBS had a significant effect.

#### NOR

MIA rats appeared to show a lower performance in NOR when tested 24 h after first presentation of objects. A comparison of MIA and control rats using *t*-test revealed a statistically significant difference (*T*_(23)_ = 2.130, *p* = 0.044, *d* = 0.85; Figure 1D). ANOVA conducted with the factors GROUP and iTBS revealed no significant effects of each factor and no interaction between both when applied to the sham- or verum-iTBS treated groups.

#### PFST and SCT

In case of PFST and SCT neither differences between MIA and control rats nor effects of iTBS were found (Figures 1E,F).

#### MWM

**Figure 2:**
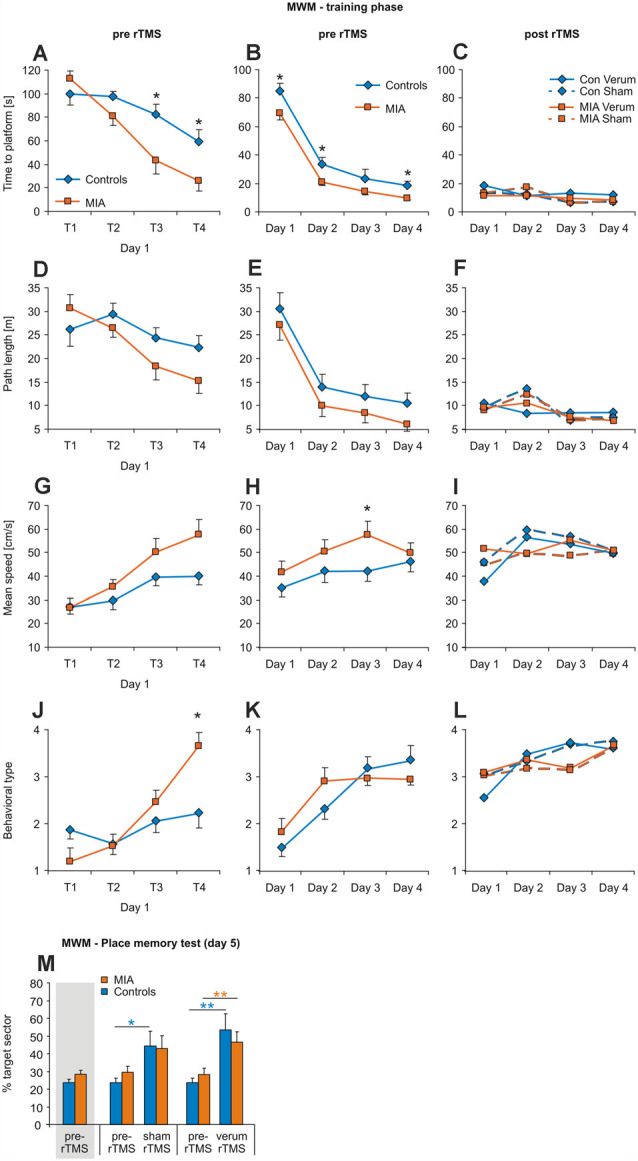
iTBS applied to adult rats: effects on morris water maze (MWM). Measures of time to platform **(A–C)**, total path length **(D–F)**, mean swimming speed **(G–I)** and behavioral type **(J–L)** are separately shown for the four trials of the first training day (left column), for the means of days 1–4 (middle column) and for the days 1–4 when repeating MWM after sham or verum-iTBS (right column) with the platform position now shifted from north to south quadrant. **(M)** Results of the place memory test conducted on day 5 with the platform removed. Shown are percent time spent within the target quadrant during the first 30 s of testing both, for the test prior to iTBS and after either verum- or sham-iTBS. Colored asterisks: paired *t*-test [controls: blue, MIA: orange, in this case pre-iTBS data shown separately for sham and verum groups but pooled in case of the leftmost columns (gray shading)], black asterisks: *t*-test for independent samples (MIA vs. controls, 2-sided). *p < 0.05, **p < 0.01. Data are shown as group means ± SEM. For clarity, SEMs are not shown for the post-iTBS samples. *n*/groups as in Figure 1.

All rats learned to find and remember the hidden platform during the first training phase prior to iTBS (days 1–4), evident by the progressive shortening of the time and the path to reach the platform when analyzed per day and for the individual trials of the first day (T 1–4). Also an increase in swimming speed and behavioral type was evident (see Figure 2). ANOVA conducted with the factors GROUP (MIA, controls) and either DAY (days 1–4) or TRIAL (trials 1–4 of day 1) revealed a significant effect of factors DAY and TRIAL for all measures (see Tables s 1–3), as could be expected. Furthermore, factor GROUP appeared to be effective for time-to-reach platform (target) and mean swimming speed when ANOVA was applied to days 1–4 (Table 2), and a significant interaction between TRAIL and GROUP was evident in case of performance type (Table 3). A pairwise comparison of MIA and control rats using *t*-test revealed better performance of MIA rats on days 1, 2 and 4 for time-to-platform (Table 2, Figure 2B) and for the trials 3 and 4 of day 1 (Table 3, Figure 2A, T3, T4). A significantly better performance of MIA rats was also evident for behavioral type on trial 4 of day 1 and a higher mean speed on day 3.

**Table 2:** ANOVA and *post hoc* *t*-test results of the analysis of Morris water maze (MWM) data in case of iTBS applied to adult rats, using factors Group (MIA, controls) and Days (1–4).

	ANOVA					*t*-test MIA vs. controls (sign. cases)				
Parameter	Factor	DF	*F*-value	*p*-value	*η*^2^	Parameter	DF	*T*-value	*p*-value	*d*
Time to target
	Day	3	76.993	**<0.001**	0.384	Day 1	22	2.149	**0.043**	0.88
	Group	1	4.897	**0.028**	0.013	Day 2	22	2.336	**0.029**	0.96
	Day × Group	3	0.1	0.960	0.001	Day 4	22	2.162	**0.041**	0.88
Path length										
	Day	3	68.915	**<0.001**	0.357					
	Group	1	2.866	0.091	0.008					
	Day × Group	3	0.198	0.898	0.002					
Mean Speed										
	Day	3	3.383	**0.018**	0.027	Day 3	22	3.464	**0.002**	1.41
	Group	1	10.074	**0.002**	0.027					
	Day × Group	3	1.565	0.198	0.013					
Perform. type										
	Day	3	34.741	**<0.001**	0.239					
	Group	1	3.192	0.075	0.01					
	Day × Group	3	0.523	0.667	0.005					

*DF, degrees of freedom; *η*^2^, partial ETA; d, Cohen’s d; *p*-values of significant cases (<0.05) are shown in bold font*.

**Table 3:** ANOVA and *post hoc* *t*-test results of the analysis of MWM data in case of iTBS applied to adult rats, using factors Group (MIA, controls) and trials (1–4).

	ANOVA					*t*-test MIA vs. controls (sign. cases)				
Parameter	Factor	DF	*F*-value	*p*-value	*η*^2^	Parameter	DF	*T*-value	*p*-value	*d*
Time to target
	Trial	3	14.063	**<0.001**	0.327	Trial 3	22	2.553	**0.019**	1.04
	Group	1	2.109	0.150	0.024	Trial 4	22	2.474	**0.022**	1.01
	Trial × Group	3	1.619	0.191	0.053					
Path length										
	Trial	3	4.780	**0.004**	0.143
	Group	1	1.190	0.278	0.014					
	Trial × Group	3	0.667	0.575	0.023					
Mean Speed										
	Trial	3	6.721	**<0.001**	0.192					
	Group	1	1.298	0.258	0.015					
	Trial × Group	3	0.711	0.548	0.024					
Perform. type										
	Trial	3	13.171	**<0.001**	0.354	Trial 4	22	2.12	**0.046**	0.86
	Group	1	2.076	0.154	0.028					
	Trial × Group	3	2.493	**0.043**	0.106					

*DF, degrees of freedom; *η*^2^, partial ETA; d, Cohen’s d; *p*-values of significant cases (<0.05) are shown in bold font*.

Following sham- or verum-iTBS, all groups started at a high level of performance for the second block of training sessions with the position of the platform now changed from north to south (Figures 2C–L). No significant differences for any kind of measure were evident between the groups MIA-sham, MIA-verum, control-sham and control-verum. Place memory test (% time in target sector, first 30 s) with the platform removed on day 5 revealed no differences between MIA and control rats prior to iTBS and performance increased both, after sham and verum-iTBS, although somewhat better after verum-iTBS (Figure 2M). ANOVA conducted with factors iTBS and GROUP resulted in a significant effect of factor iTBS only (*F*_(2,42)_ = 12.697; *p* < 0.001; *η*^2^ = 0.377) without interaction between these factors. *Post hoc* paired *t*-test indicated a significant increase in time spent in target sector after sham- (*T*_(6)_ = −2.931, *p* = 0.033, *d* = 1.29) and verum-iTBS (*T*_(6)_ = −4.040, *p* = 0.009, *d* = 1.71) for the controls and a significant increase after verum-iTBS (*T*_(6)_ = −3.388, *p* = 0.01, *d* = 1.31) in case of the MIA rats.

### Exp. Series II: iTBS Applied to Juvenile Rats (Preventive Approach)

Application of iTBS to juvenile MIA rats aimed at testing a possible preventive iTBS effect. Therefore, rats received 2 weeks of daily iTBS (Monday–Friday, 3 blocks/day, either sham or verum-iTBS) at an age of 6 weeks without prior behavioral testing. Behavioral testing was conducted when the rats were 12–13 weeks old. This experimental series was conducted in seven blocks with litters of MIA and control rats randomly assigned to the sham or verum-iTBS groups (all male), meaning that each of the four groups included offspring cumulating from seven different litters (*n* = 12–14).

#### PPI

**Figure 3:**
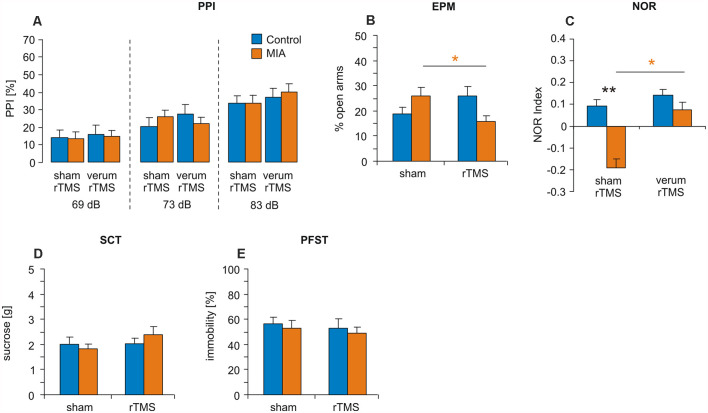
iTBS applied to juvenile rats: effects on PPI, EPM, NOR, SCT and PFST. MIA and control rats were tested at adulthood for **(A)** PPI, **(B)** EPM, **(C)** NOR, **(D)** SCT and **(E)** PFST after iTBS-rTMS (sham or verum) had been applied during adolescence. Colored asterisks: *t*-test comparing sham and verum-iTBS groups (controls: blue, MIA: orange), black asterisks: *t*-test comparing MIA and control groups. *p < 0.05, **p < 0.01. Data are shown as group means ± SEM. *N* = 12 control-sham, *N* = 12 control-verum, *N* = 13 MIA-sham, *N* = 14 MIA-verum.

PPI test revealed no significant differences between the four experimental groups when tested with prepulse intensities of 69 dB, 73 dB and 83 dB (Figure 3A). Factors GROUP (MIA, Controls) and iTBS (sham, verum) tested with ANOVA appeared to be both ineffective and without interaction.

#### EPM

EPM test revealed no significant effects of factors GROUP and iTBS when tested with ANOVA but a significant interaction between these factors (*F*_(1,47)_ = 7.520, *p* = 0.009, *η*^2^ = 0.143). A pairwise comparison of MIA vs. control groups (Figure 3B) and sham vs. verum-iTBS groups revealed a significant difference between sham- and verum-iTBS treated MIA groups (*T*_(22)_ = 2.520, *p* = 0.018, *d* = 0.99).

#### NOR

NOR test conducted with 24 h interval showed poor performance of MIA rats compared to controls (Figure 3C). ANOVA with factors GROUP and iTBS revealed significant effects of both factors but without interaction (GROUP: *F*_(1,47)_ = 6.998, *p* = 0.011, *η*^2^ = 0.13; iTBS: *F*_(1,47)_ = 5.615, *p* = 0.022, *η*^2^ = 0.11). Pairwise comparison indicated a significant difference between MIA and control groups subjected to sham-iTBS (*T*_(23)_ = 2.910, *p* = 0.008, *d* = 1.16) and between sham- and verum-iTBS treated MIA rats (*T*_(25)_ = 2.612, *p* = 0.015, *d* = 1.00).

#### PFST and SCT

Also in case of the rats treated with iTBS during adolescence no differences between MIA and control rats and no effects of iTBS were evident for PFST and SCT (Figures 3D,E).

#### MWM

**Figure 4:**
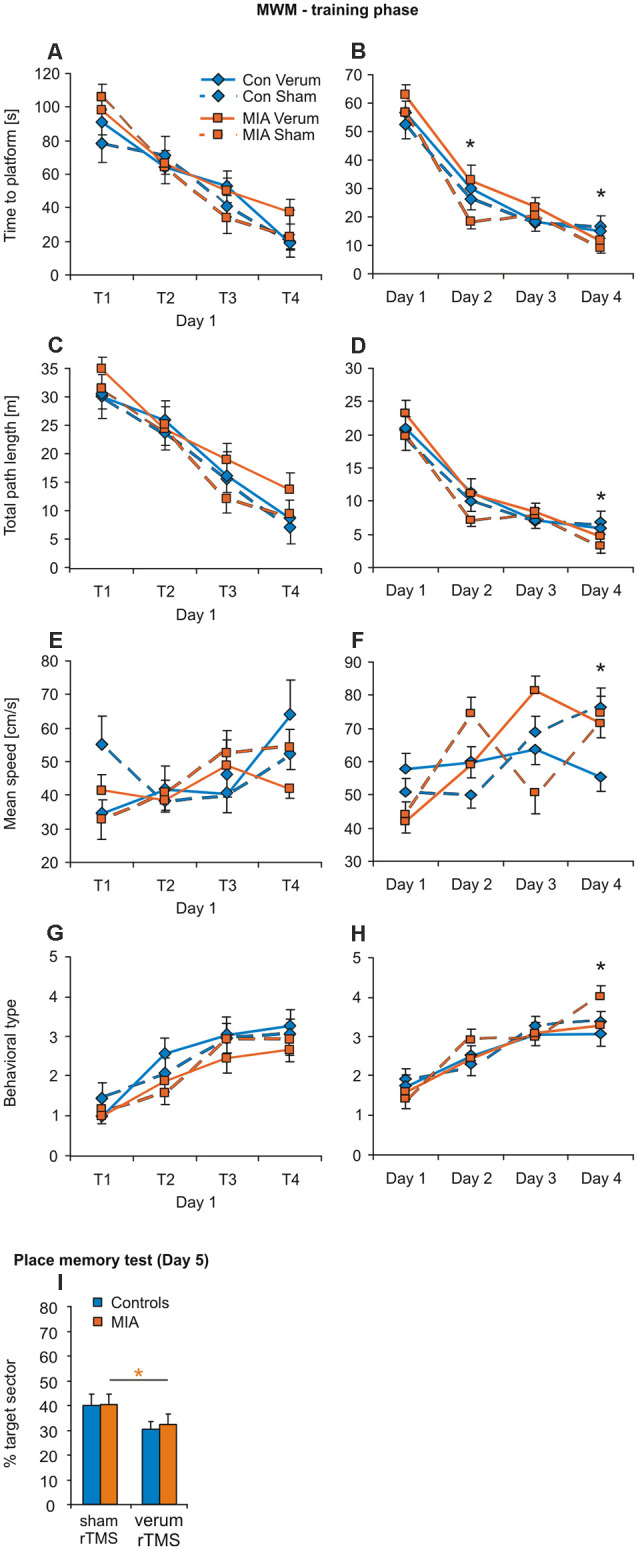
iTBS applied to juvenile rats: effects on MWM. Training phase of MWM with the trials of day 1 **(A,C,E,G)** separately shown from the means of the trials for day 1–4 **(B,D,F,H)** for the measures: time to platform, total path length, mean swimming speed and behavioral type. **(I)** Results of the place memory test conducted on day 5 with the platform removed. Shown are percent time spent within the target quadrant during the first 30 s of testing. Colored asterisks: *t*-test comparing sham vs. verum-iTBS groups (controls: blue, MIA: orange), black asterisks: *t-test* comparing sham-iTBS treated MIA and control groups. *p < 0.05. Data are shown as group means ± SEM. *N* = 12 control-sham, *N* = 12 control-verum, *N* = 13 MIA-sham, *N* = 14 MIA verum.

No principal difference in learning performance was evident between the four groups during the training phase (Figures 4A–H). ANOVA conducted with factors GROUP, iTBS and either TRIALS (trials 1–4 of the first day), or DAYS (days 1–4), revealed only factors TRIALS and DAYS as being effective in any case of measure (see Tables s 4, 5). However, a pairwise comparison of the data of the MIA and control groups revealed a better performance of MIA rats with measure time-to-platform (target) at days 2 and 4 (4). Furthermore, MIA rats showed better performance in path length, mean speed and behavioral type on training day 4 (Table 4). No differences between the four groups were evident for the first four trials on day 1.

**Table 4:** ANOVA and *post hoc* *t*-test results of the analysis of MWM data in case of iTBS applied to juvenile rats, using factors Group (MIA, controls) and Days (1–4).

	ANOVA					*t*-test MIA vs. controls (sign. cases)				
Parameter	Factor	DF	*F*-value	*p*-value	*η*^2^	Parameter	DF	*T*-value	*p*-value	*d*
Time to target
	Day	3	32.796	**<0.001**	0.384	Day 2	23	2.672	**0.009**	0.59
	Group	3	1.184	0.315	0.013	Day 4	23	2.239	**0.028**	0.37
	Day × Group	9	0.748	0.665	0.001					
Path length
	Day	3	99.787	**<0.001**	0.272	Day 4	23	2.323	**0.022**	0.46
	Group	3	2.294	0.077	0.009					
	Day × Group	9	0.913	0.513	0.010					
Mean Speed
	Day	3	8.730	**<0.001**	0.033	Day 4	23	2.134	**0.035**	0.45
	Group	3	0.976	0.404	0.004					
	Day × Group	9	1.431	0.170	0.017					
Perform. type
	Day	3	69.950	**<0.001**	0.223	Day 4	23	2.362	**0.020**	0.48
	Group	3	0.613	0.606	0.003					
	Day × Group	9	1.001	0.437	0.012					

*DF, degrees of freedom; *η*^2^, partial ETA; d, Cohen’s d; *p*-values of significant cases (<0.05) are shown in bold font*.

**Table 5:** ANOVA results of the analysis of MWM data in case of iTBS applied to juvenile rats, using factors Group (MIA, controls) and trials (1–4).

Parameter	Factor	DF	*F*-value	*p*-value	*η*^2^
Time to target					
	Trial	3	14.063	**<0.001**	0.327
	Group	1	2.109	0.150	0.024
	Trial × Group	3	1.619	0.191	0.053
Path length					
	Trial	3	4.780	**0.004**	0.143
	Group	1	1.190	0.278	0.014
	Trial × Group	3	0.667	0.575	0.023
Mean Speed					
	Trial	3	6.721	**<0.001**	0.192
	Group	1	1.298	0.258	0.015
	Trial × Group	3	0.711	0.548	0.024
Perform. type					
	Trial	3	13.171	**<0.001**	0.354
	Group	1	2.076	0.154	0.028
	Trial × Group	3	2.493	**0.043**	0.106

*DF, degrees of freedom; *η*^2^, partial ETA; *p*-values of significant cases (<0.05) are shown in bold font*.

In case of final place memory test on day 5 (Figure 4I), no difference was found between MIA and control rats for the time spent in the target sector (first 30 s) but verum-iTBS treated groups showed a lower performance than sham-treated groups. Only factor iTBS but not factor GROUP tested with ANOVA appeared to be effective (*F*_(1,53)_ = 8.955, *p* = 0.004, *η*^2^ = 0.145). *T*-test revealed a significant difference between sham- and verum-iTBS treated MIA rats (*T*_(25)_ = 2.091, *p* = 0.047, *d* = 0.80).

## Discussion

### Summary of Findings—Differences Between MIA Offspring and Controls

The primary goal of this study was to test the potential of iTBS to treat the behavioral symptoms of schizophrenia either as a therapeutic tool applied during adulthood when the pathological phenotype is already established, or in a preventive fashion during development of the juvenile brain. For this reason, we had chosen the rat MIA model of schizophrenia because it is mainly based on developmental perturbations allowing possible modulation of misbalanced neuronal networks. Behavioral tests comprised PPI, the “gold standard” to test deficits in sensory gating in animal models of schizophrenia (Swerdlow et al., 2008) complemented by standard testing of anxiety, depression, anhedonia and cognitive performance (e.g., see Bergdolt and Dunaevsky, 2019) because iTBS had not been tested before on these behavioral phenotypes and because schizophrenia and MIA are often associated with anhedonia and depressed states (Buckley et al., 2009; Pelizza and Ferrari, 2009). Our data show that the MIA offspring perform better during the training phase of MWM, which had not been described so far, and confirm previously described deficits of MIA rats in sensory gating (PPI; Wolff and Bilkey, 2008; Hadar et al., 2015) and NOR performance (Ito et al., 2010; Wolff et al., 2011; Gray et al., 2019; Saunders et al., 2020). Most studies testing classical MWM found no effects of MIA on performance with regard to acquisition and remembering the platform location (Zuckerman and Weiner, 2005; Piontkewitz et al., 2009; Vorhees et al., 2015). Interestingly, Wolff and Bilkey (2015) reported that, compared to controls, MIA offspring exhibit smaller receptive fields of hippocampal place cells, indicating that local spatial orientation may be increased but contextual orientation requiring integration of a wider space may be impaired (Wolff et al., 2011).

Reports on anxiety-related behavior of MIA offspring are contradictory with some describing increased anxiety (Shi et al., 2003; Abazyan et al., 2010; Yee et al., 2011; Canetta et al., 2016) while others found no difference (Ratnayake et al., 2012; Li et al., 2014; Vorhees et al., 2015) or even reduced anxiety (Ozawa et al., 2006). Our tests revealed no differences between MIA and controls rats in EPM test, neither if tested in adult rats prior to iTBS, nor after having received iTBS during adolescence. We further found no signs of depression, like anhedonia or early surrender in PFST, as has been described as a possible co-morbidity of schizophrenia and a further consequence of MIA (Samsom and Wong, 2015). However, specific changes of the behavioral phenotype in MIA offspring are highly dependent on the kind and timing of interference of genetic and environmental factors (see Missault et al., 2014; Ronovsky et al., 2016).

Like others, our study confirms that MIA offspring are primarily impaired in behavior requiring attentional decision as with PPI and NOR. Canetta et al. (2016) and Wallace et al. (2014) demonstrated MIA offspring being deficient in attentional set shifting and reversal learning (Wallace et al., 2014) but no signs of deficits in working memory were evident (Canetta et al., 2016; Nakamura et al., 2021), the latter being in accordance with the lack of deficits we found with MWM test. Canetta et al. (2016) further demonstrated an impaired GABAergic transmission in the mPFC of adult MIA offspring which was selective for PV-expressing interneurons. This class of interneurons has gained interest as they are essential for the generation of cortical oscillations in the gamma frequency (30–80 Hz; Cardin et al., 2009), assumed to support cognitive processes including working memory and attentional set shifting (Tallon-Baudry et al., 1998; Uhlhaas and Singer, 2010; Cho et al., 2015).

### Summary of Findings—Effects of iTBS at Adolescence or Adulthood

According to the changes in oscillatory activity we observed after of iTBS in MIA rats (Lippmann et al., 2021) we also expected to find changes at the behavioral level. Deficits in PPI declined if MIA rats received iTBS at adulthood. However, sham-iTBS was as effective as verum-iTBS, indicating little or no *specific* effects of the brain stimulation itself. Surprisingly, control rats showed a clear decrease in PPI following sham and verum-iTBS while the MIA rats improved. MIA offspring receiving iTBS during adolescence did not show PPI values different from control rats, regardless of verum or sham treatment, a further indication that sham and verum-iTBS were similarly effective. However, for unknown reasons, these rats exhibited generally lower PPI values (ca. −20%, MIA and controls) compared to adult control rats not being stimulated before. It cannot be excluded that early experiences deviating from the conventional housing conditions may alter the rat’s behavioral phenotype as determined by standard testing. This impression is supported by the findings of Kentner et al. (2016) and Zhao et al. (2020), demonstrating that housing MIA offspring in an enriched environment (EE) reverses the cognitive deficits in spatial discrimination and the down-regulation of genes critical to synaptic transmission and plasticity. Similarly, rodent studies using NMDA receptor antagonist dizolcilpine (MK-801) injection to induce a schizophrenia-like state demonstrated beneficial effects of EE, reversing the MK-801 induced deficits in NOR and PPI (Murueta-Goyena et al., 2018; Huang et al., 2021), accompanied by recovery of PV immunoreactivity. Interestingly, the same effect could be achieved by selective chemogenetic activation of PV-expressing interneurons within frontal association cortex (Huang et al., 2021). On the other hand, a study investigating the effects of an enriched environment on the behavior of normal Wistar rats in EPM, radial maze, operant conditioning, ASR and PPI showed no clear differences to rats housed at standard conditions (Hoffmann et al., 2009), indicating that experience of an enriched environment is not directly comparable with the experience of testing and handling. However, it cannot be excluded that MIA offspring react in a different way than normal rats.

On the other hand, a specific stimulation effect was evident for NOR if iTBS had been applied during adolescence, since improvement was evident only after verum-iTBS. Interventions modulating cortical excitability and the interplay of excitatory and inhibitory synaptic activities appear to be effective if applied to the juvenile brain (Huang et al., 2021). Of note, phenotype specific, i.e., pathology dependent effects of NIBS during adolescence have previously been reported both in the MIA (Hadar et al., 2020) and the dopamine transporter overexpressing rat (DAT+) model of repetitive symptoms (Edemann-Callesen et al., 2018). tDCS of frontal cortex prevented the schizophrenia-like symptoms of MIA rats when applied during adolescence and ameliorated repetitive symptoms when applied to adult DAT+ rats (Edemann-Callesen et al., 2018). We recently described that MIA rats show disturbed long-range synchrony of neuronal theta-oscillations, in particular between medial prefrontal cortex (mPFC) and ventral hippocampus, aberrant prefrontal gamma-theta phase coupling and an overall increase in the ratio of low to high frequency oscillations of brain activity (Lippmann et al., 2021). rTMS with iTBS protocol and deep brain stimulation (DBS) within mPFC were able to normalize these activity patterns at least acutely. Similar disturbances of limbic brain oscillations have been observed in psychiatric diseases including schizophrenia and are assumed to be a neuronal counterpart of cognitive deficits related to malfunction of attention (see Leicht et al., 2016; Northoff and Duncan, 2016; Hunt et al., 2017). Hence, deficits in sensory gating (PPI) and NOR as found in the MIA rats, and also the partial improvement of both measures following iTBS well match the effects on oscillatory limbic brain activity as described above.

### Sham-iTBS Effects vs. Re-test Effects and Differences Between MIA and Control Rats

Results obtained for PPI, EPM and in part NOR revealed strong changes when adult rats were re-tested after iTBS, not only after verum but also after sham-iTBS. Since classical placebo effects as in humans can be excluded, the history of handling and testing appears to be a relevant factor, in particular when re-testing animals with the same paradigm. The decrease in time spent on the open arms of the EPM after iTBS could be interpreted as increased anxiety. However, others discuss the reduced open arm visits in the re-test condition as less exploratory drive due to previous experience (see Carobrez and Bertoglio, 2005). In case of PPI, no data on re-test effects appear to be available to separate re-test effects from effects related to the iTBS procedure when tested under sham condition. Remarkably, clear differences between MIA and control rats were evident when re-tested after sham-iTBS, indicating that the experimental experiences affected the behavioral phenotype of MIA and control rats differently, and obviously more than verum stimulation: PPI performance significantly increased in case of MIA rats but decreased in controls after sham-iTBS. And, open arm stays in EPM generally decreased after sham-iTBS but significantly more in controls.

The significance of the history of experiences has been well documented not only for the after-effects of rTMS in humans (Ridding and Ziemann, 2010; Smith et al., 2010; Karabanov et al., 2015; Suppa et al., 2016). Donato et al. (2013) demonstrated that, compared to non-fearful experiences (enriched environment), a fearful experience (foot-shock fear conditioning) weakens the performance of rats in subsequent cognition tests. Since changes in the activity of hippocampal inhibitory interneurons expressing the calcium-binding protein PV are involved in this process and are also considered in the pathology of psychiatric diseases (Chung et al., 2016), MIA and control rats likely differ also in the way of experience-dependent responses (Canetta et al., 2016). As one limitation of our study we have to submit that an additional group of adult animals receiving neither verum- nor sham-iTBS would have be useful to clarify whether not only repeated testing but also the procedures of applying rTMS influence the behavior. However, given that real placebo effects due to expectations in the procedure can be excluded, we decided to omit such a group. Nevertheless, future studies of this kind may consider such an additional control group.

## Final Conclusions

Our study aimed at investigating the possible therapeutic and preventive effect of iTBS applied with iTBS protocol in a rat schizophrenia model. It turned out that verum-iTBS had little specific beneficial effect related to changes in behavioral phenotype. If applied during adolescence, improvement was found only for NOR. Improvement in sensory gating (PPI) of MIA rats—but worsening performance of controls—was evident both, after verum- and sham-iTBS applied in a therapeutic manner, indicating that effects of environmental factors are superior to iTBS effect in the Wistar rats used in this study and, most importantly, affect MIA and control rats differently. In translational terms these findings support the importance of experiences, like physical exercise, social and cognitive training and even enriched environments, as adjunct therapeutic interventions in treating schizophrenia symptoms in humans (Spielman et al., 2016; Girdler et al., 2019; Gómez-Rubio and Trapero, 2019; Maurus et al., 2019) besides the hopeful experiences originating from the care by a medical doctor and the clinical facilities.

## Data Availability Statement

The original contributions presented in the study are included in the article, further inquiries can be directed to the corresponding author.

## Ethics Statement

The animal study was reviewed and approved by State Office for Nature, Environment and Consumer Protection, LANUV, Section 81-Animal Welfare, Az. 84-02.04.2014.A294.

## Author Contributions

KF, CW and VA designed the experiments. NR, TI, GB and VA conducted the experiments. NR, TI, VA and KF did the data analysis. KF wrote the first draft of the manuscript. All authors contributed to the article and approved the submitted version.

## Conflict of Interest

GB was employed by the company AIO-Studien-gGmbH. The remaining authors declare that the research was conducted in the absence of any commercial or financial relationships that could be construed as a potential conflict of interest

## Abbreviations

ANOVA: analysis of variance
ASR: acoustic startle response
DAT+: dopamine transporter overexpressing rat
DBS: deep brain stimulation
EPM: elevated plus maze
GD: gestational day
iTBS: intermittent theta-burst stimulation
MIA: maternal immune activation
MK-801: NMDA receptor antagonist dizolcilpine
mPFC: medial prefrontal cortex
MWM: Morris water maze
NIBS: non-invasive brain stimulation
NOR: novel object recognition
PFST: Porsolt forces swim test
PolyI:C: polyinosinic:polycytidylic acid
PPI: prepulse inhibition
PV: parvalbumin
rTMS: repetitive transcranial magnetic stimulation
SCT: sucrose consumption test
tDCS: transcranial direct current stimulation.
